# CpG-ODN Facilitates Effective Intratracheal Immunization and Recall of Memory against Neoantigen-Expressing Alveolar Cells

**DOI:** 10.3389/fimmu.2017.01201

**Published:** 2017-09-29

**Authors:** Mathias Riehn, Marcin Cebula, Hansjörg Hauser, Dagmar Wirth

**Affiliations:** ^1^Research Group Model Systems for Infection and Immunity, Helmholtz Centre for Infection Research, Braunschweig, Germany; ^2^Division of Experimental Hematology, Hannover Medical School, Hannover, Germany

**Keywords:** neoantigen, cytotoxic T cells, tolerogenic, alveolar tract, immune modulation

## Abstract

Intrapulmonary immune reactions are impaired by the tolerogenic environment of the lung. This is manifested by the absence of effective endogenous T cell responses upon neoantigen expression. This tolerance is considered to contribute to lung cancer and inefficient immune therapeutic interventions. To investigate the mechanisms contributing to lung tolerance and to overcome these restrictions, we developed a transgenic mouse model with induction of a neoantigen (OVA) exclusively in alveolar type II epithelial cells. This model is characterized by the absence of functional endogenous T cell responses upon OVA neoantigen induction. Standard DNA and protein vaccination protocols resulted in the accumulation of high numbers of antigen-specific CD8 T cells in the lung. However, clearance of antigen-expressing cells was not achieved. To overcome this tolerance, we induced inflammatory conditions by coapplication of the TLR ligands LPS and CpG-ODN during intrapulmonary vaccinations. Both ligands induced high numbers of neoantigen-specific T cells in the lung. However, only coapplication of CpG-ODN was sufficient to establish functional cytotoxic responses resulting in the elimination of neoantigen presenting target cells. Remarkably, CpG-ODN was also crucial for functional memory responses upon re-induction of the neoantigen. The results highlight the need of TLR9 co-stimulation for overcoming tolerization, which might be a key factor for therapeutic interventions.

## Introduction

The lung epithelium represents an interface that is constantly exposed to environmental pathogens such as bacteria, fungi, and viruses. At the same time, it is confronted with myriads of harmless airborne particles that provide huge amounts of antigenic material. Constitutively expressed proteins such as lysozyme, defensins, and lactoferrin as well as infections induce the release of type I interferons constituting the immediate innate response against pathogens. Later, in a second line, the adaptive immune response sets in which includes the formation of neutralizing antibodies and antigen-specific T lymphocytes. Among the various immune cells in lung contributing to combat respiratory infections, cytotoxic T cells are critical mediators of the adaptive immune system. Upon encounter of a cognate antigen, these cells acquire effector functions, express cytokines such as IFNγ and TNFα, and induce cell death.

To avoid overwhelming immune responses to high level of extrinsic neoantigen and the associated tissue damage, the activity of cytotoxic T cells in the lung has to be tightly controlled. This so-called tolerogenic environment in the lung is established by different mechanisms.

Soluble factors are discussed to contribute to the establishment of such a tolerogenic environment in the lung. Indeed, tumor-associated macrophages in lung tumor produce large amounts of TGF-β that can block cytotoxic T cell function (1). In this regard, alveolar macrophages are considered to contribute to a niche for tumor development (2). Also, other cell-based mechanisms contribute to the tolerogenic environment in the lung. This particularly concerns the development of regulatory T cells, which are fostered by dendritic cells (3, 4) and by alveolar type II epithelial cell (AECII) (5), in steady state conditions. Remarkably, in the situation of infection, alveolar macrophages (6) and dendritic cells (7–9) can shift their immune suppressive phenotype to a pro-inflammatory phenotype. This is mediated upon sensing the pathogens *via* pattern recognition receptors (PRRs) like TLR4 or TLR9 which initiate, e.g., by the adapter protein MYD88, the intracellular NF-κB pathway with subsequent production of pro-inflammatory cytokines (10).

Viral antigen presentation during acute infection is associated with activation of cellular PRRs by pathogen-associated molecular patterns (PAMPs). This coactivation of innate defense mechanisms is considered to contribute to efficient immune responses. As a consequence, in sterile conditions when PAMPs are absent, immune responses are considered to be suboptimal. Such pathological situations can arise in chronic infections in which pathogens hide within the cell and PAMPs are not detected but also in cancer where genetic rearrangements lead to expression of modified or mutated proteins, which represent new antigenic structures, so-called neoantigens (11, 12).

In the recent years, efforts to improve immune-based approaches against cancer and chronic infections made substantial progress. Therapeutic vaccination can be employed to instruct the endogenous immune system. Presentation of neoantigens by professional cells is sufficient for the establishment of neoantigen-specific T cells (13). Still, various studies show the inefficiency of such vaccination-induced T cells. In these instances, the cytotoxicity of T cells is reduced, and an exhausted phenotype is established. Thus, strategies have been developed to rescue exhausted T cells by blocking immune checkpoints that control and impair proper T cell activation [such as programmed cell death protein 1 (PD-1) or CTLA-4 pathway] (14–18). While these strategies have been shown to improve immune-based strategies against cancer and chronic infections, their clinical translation is not always straightforward since, e.g., only about 20–25% of non-small cell lung cancer patients profit from such treatments (19).

Another strategy to improve T cell responses in sterile conditions is to artificially provide PAMPs to stimulate the PRRs. PRRs, including toll-like receptors, are expressed by different immune cells, most prominently by professional antigen presenting cells, and modulate the local immune response of T cells either directly or indirectly (20). The relevance of TLR signaling for establishing a potent immune response has been harnessed for vaccine development. Accordingly, various TLR ligands are considered as potent adjuvants in vaccine protocols (21).

Immune responses in the lung are usually investigated in infection models that rely on pathogen-delivered neoantigens. Such models are accompanied by inflammation including strong activation of TLR pathways and thus do not reflect the conditions in early steps of tumor development in which antigen presentation occurs in the infection-free environment. Similar obstacles come with transfer of neoantigen-expressing tumor cells, since injection of cells is associated with debris and other cell compounds able to trigger PRRs. In this regard, transgenic animals with transcriptionally or genetically controlled neoantigen expression represent an attractive tool to investigate the *de novo* response to neoantigens under sterile conditions. However, they require tight control of antigen expression. Leakiness would result in the establishment of peripheral tolerance and lack of neoantigen recognition. Here, we present a novel model for inducible antigen presentation in AECII cells relying on Tamoxifen-induced, Cre-mediated recombination that leads to specific neoantigen expression in lung. We show that vaccination-induced T cells expand and populate the lung. Interestingly, TLR9, but not TLR4, stimulation is crucial for activating a potent cytotoxic T cell activity in the lungs. Moreover, we give evidence that an impaired memory response to reoccurring neoantigen can be restored by stimulation by CpG-ODN.

## Materials and Methods

### Transgenic Mice

The transgenic SpcCreOVA mice employed in the study have the C57BL6/J genetic background and have been created by breeding ROSAOVA mice (22) with SpcCreERT2 mice (23). In brief, the SpcCreOVA mice carry an inactive synthetic OVA gene cassette flanked by inversely oriented LoxP sites integrated into the ubiquitously expressed ROSA26 locus. Tamoxifen-induced Cre recombination and inversion of the cassette activates of OVA expression in AECII. Mice were maintained and bred in individually ventilated cages under specific pathogen-free conditions.

### Animal Treatment

For neoantigen induction, 6- to 12-week-old mice were force-fed by gavage with 1 mg Tamoxifen (ALIUD Pharma GmbH & Co. KG) in 200 µl Clinoleic (Baxter Healthcare) per 25 g mouse (if not indicated differently). For intratracheal vaccination, mice were narcotized with Ketamin/Xylazine (intraperitoneal), at least 2 weeks after Tamoxifen feeding. For intratracheal administration, the TLR4 ligand LPS (Sigma, St. Louis, MO, USA) (0.25 mg/kg), the TLR9 ligand CpG-ODN1668: 5′-S-TCCATGACGTTCCTGATGCT-3′ (TIB Molbiol, Berlin, Germany) (1.2 mg/kg), and OVA protein (Serva, Heidelberg, Germany) (1.2 mg/kg), was used either alone or in combination as specified in the figures. In all applications, the ligands and/or OVA were solved in 50 µl sterile PBS and applied for intratracheal administration. For the boost, vaccination procedure was repeated, but with LPS dose increased to 0.75 mg/kg.

### T Cell Isolation

Isolated lungs were cut into pieces; lung tissue was digested in 1 mg/ml Collagenase D (Roche; Basel, Switzerland) and 0.5 mg/ml DNAse I (Roche, Basel, Switzerland) for 45 min under constant shaking at 37°C. After passing the digested tissue through a 100 µm cell strainer (Corning, Corning, NY, USA) in PBS with 2% FCS, the remaining erythrocytes were lysed with ACK buffer (NH_4_Cl 150 mM, KHCO_3_ 10 nM, EDTA 0.1 nM). After subsequent washing steps, cells were stained and analyzed by flow cytometry.

### Flow Cytometry

Isolated T cells were stained in PBS with 2% FCS. Following fluorochrome-labeled antibodies were used: αCD8-Percp/Cy5.5 (eBioscience/BioLegend, clone: 53-6.7), αCD4-eFlour450 (eBioscience, clone: Gk1.5), αCD62L-PE-Cy7 (eBioscience, clone: MEL-14), αCD69-FiTC (eBioscience/Biolegend clone: H1.2F3), CD44-APC (eBioscience clone: IM7), αCD19-APC-Cy7 (BD clone: 1D3), αCD223/Lag-3-APC (eBioscience clone: eBioC9B7W), αCD279/PD-1-FiTC (eBioscience clone: J43), αCD3e-FiTC/PE (eBioscience clone: 145-2C11), H-2kb-SIINFEKL-PE [ProImmune, Batch: PP(6080-2)]. To assess the effector cytokine production in OVA-specific T cells, isolated cells were restimulated for 7 h with SIINFEKL peptide. For the last 5 h of restimulation, Brefeldin A (eBioscience) was added to block cytokine secretion. After surface marker staining, harvested cells were fixed and permeabilized using the BD Cytofix/Cytoperm kit (BD Bioscience). Further, the cells were subjected to intracellular staining using αTNFα-APC (eBioscience clone: MP6-XT22), and αIFNγ-FiTC (BD clone: 554411).

The proliferation of transferred OT-I cells was traced by the loss of CFSE dye after initial staining with 2.5 µM CFSE. Analyses were performed using FACS LSRII (Becton Dickinson) and FlowJo-software (TriStar Inc., USA).

### RNA Isolation

RNA was isolated out of lung biopsies of the right lung lobe using the RNeasy Minikit (Qiagen; Hilden, Germany). For reverse transcription, 2 µg RNA was used with RevertAid First Strand cDNA synthesis kit (ThermoScientific, Waltham, MA, USA), or Ready-To-Go You-Prime First-Strand Beads kit (GE; Boston, MA, USA). qRT-PCR was performed like described before (24). Primers specific for sense orientation of OVA expression cassette were used (AAGAGTCAAATGGCTCTCCTCAAGCGTATT and GTCTGTTGTGCCCAGTCATAGCCGAATAG). OVA expression was related to expression of lung tissue-specific surfactant protein C (Spc) gene (CACCATCGCTACCTTTTCCA and CTCGGAACCAGTATCATGCC). As indicated, in some experiments GAPDH was used as reference housekeeping gene [primers provided in RevertAid First Strand cDNA synthesis kit (ThermoScientific, Waltham, MA, USA)].

### Statistical Evaluation

Statistical analyses were performed with Graphpad Prism 5 and 6. Since Gaussian distribution could not be assumed *p* values were calculated by Mann–Whitney *U*-test.

## Results

### Absence of Endogenous CD8 T Cell Responses to Sterile Antigen Induction on AECII

**Figure 1:**
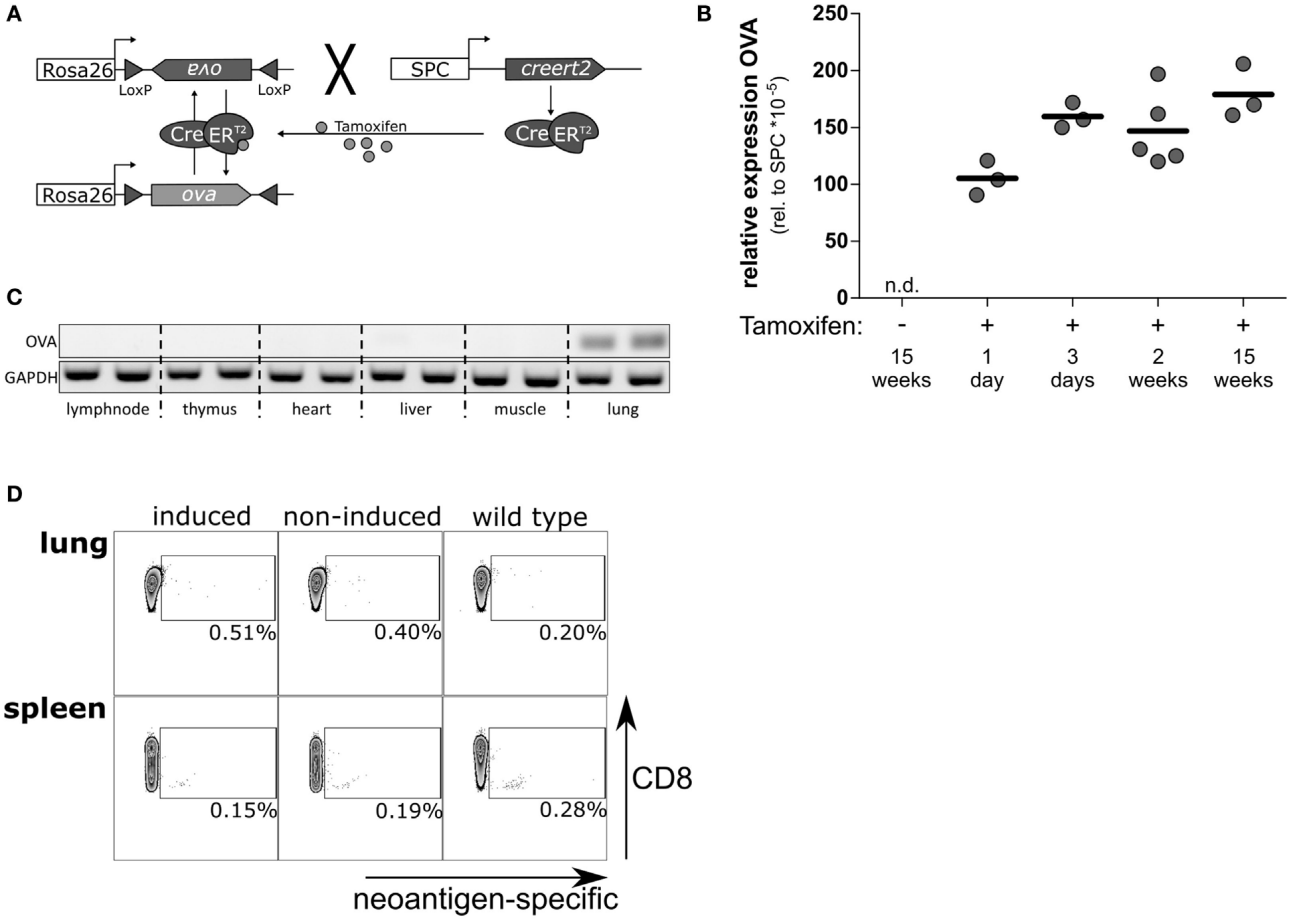
Tamoxifen-induced, lung-specific neoantigen expression in the lung is ignored by the endogenous T cells. **(A)** Schematic representation of SpcCreOVA mice. OVA coding sequence is flanked by inversely oriented loxP sites and inserted in reverse orientation downstream of the ubiquitously expressed Rosa26 promoter. CreERT2 recombinase is controlled by the Spc promoter, which is activated in AECII cells. Upon single administration of Tamoxifen, CreERT2 can shuttle to the nucleus and revert the OVA cassette, thereby activating antigen expression in AECII cells. As long as Tamoxifen is present, the cassette will undergo continuous inversion. Once Tamoxifen is cleared, the cassette is fixed, and antigen expression is achieved in about 50% of AECII. **(B)** The expression levels of OVA neoantigen in lungs of SpcCreOVA mice were determined by qRT-PCR at indicated time points after application of 1 mg Tamoxifen. The expression level of OVA was related to the expression of Spc; one representative experiment out of two experiments is shown. n.d., not detected. **(C)** OVA expression in indicated organs was assessed by RT-PCR. The RNA was isolated 2 weeks after feeding with 8 mg TAM. GAPDH was determined as control. **(D)** T cells from Tamoxifen-induced and non-induced SpcCreOVA mice, as well as wild-type mice, were isolated 2 weeks after Tamoxifen feeding. The number of neoantigens-specific CD8 T cells was determined by flow cytometry upon staining intrapulmonary T cells with pentamers directed against OVA. Depicted is one representative dot blot out of three mice per group from two independent experiments.

To investigate the T cell response to neoantigen expressed and presented under sterile conditions in the alveoli of the lung, we established the novel mouse model SpcCreOVA. To this end, we crossed RosaOva mice in which a silent, Cre-activatable gene for intracellular Ovalbumin (OVA) antigen expression is placed downstream of the ubiquitous Rosa26 promoter (22) with SpcCreERT2 mice in which Tamoxifen-dependent CreERT2 is controlled by the AECII-specific Spc promoter (23) (Figure 1A). In the absence of Tamoxifen, SpcCreOVA mice do not express detectable levels of OVA in the lung similar to wild-type mice, while single application of Tamoxifen results in the induction of OVA expression in the lung in a dose-dependent manner (Figure 1B; Figure S1 in Supplementary Material). Due to the orientation of the loxP sites, flanking the antigen-cassette, antigen expression is mosaic and is either on or off, as previously shown in the liver (25). Of note, OVA expression is restricted to the lung and could not be detected in other tissues including thymus (Figure 1C). Together, this indicates that Tamoxifen-induced OVA expression is tightly controlled and induced as a neoantigen.

We asked if the expression of OVA as neoantigen in lungs of SpcCreOva mice provokes a specific endogenous T cell response. Thus, we isolated CD8 T cells from lung and spleen on day 14 after a single administration of Tamoxifen. Isolated T cells were stained with fluorescently labeled pentamers restricted to the SIINFEKL-specific T-cell receptor. Non-induced SpcCreOVA mice and wild-type mice were used as controls. Interestingly, we could not detect a significant increase of OVA-specific CD8 T cells in lung and spleen of SpcCreOVA mice 2 weeks after induction of antigen expression in AECII (Figure 1D). The absence of a CD8 T cell response was in line with unaltered levels of OVA expression in lung even 15 weeks after the induction of antigen expression, indicating the absence of any cytotoxic activity against antigen-expressing cells (Figure 1B). To confirm that expression and presentation of the OVA epitope in AECII can be recognized by naïve T cells, we established triple transgenic SpcCreOVA X OTI mice. In these animals, most of the endogenous CD8 T cells express the OVA-specifc transgenic T cell receptor. Induction of SpcCreOVA X OTI mice with Tamoxifen-induced detectable OVA expression within 24 h. This was associated with a fast and transient accumulation of T cells in the lung, as shown by histology (Figure S2A in Supplementary Material). On day 7, elimination of OVA expression was detected in lung indicating that the OT-I cells exerted cytotoxicity (Figure S2B in Supplementary Material). This confirms the presence of a potent antigen-specific response and the immunological relevance of OVA-expressed epitope.

### High Numbers of Activated Antigen-Specific T Cells Clear Antigen-Expressing AECII

**Figure 2:**
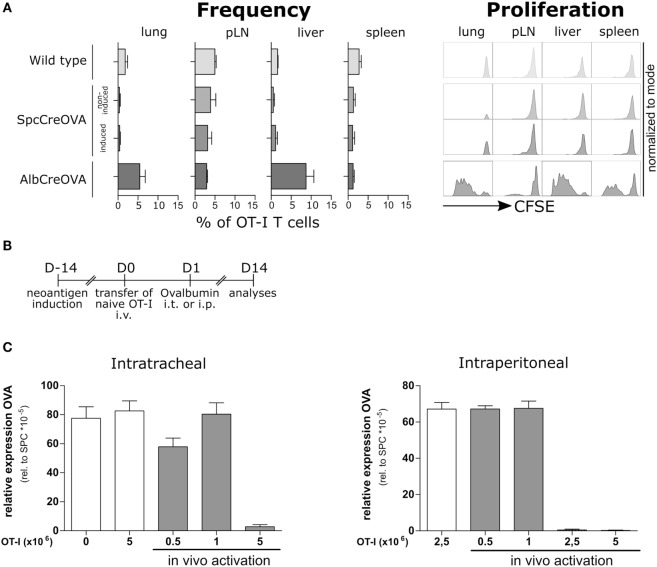
Elimination of neoantigen expressing cells by the activated, but not by naïve adoptively transferred antigen-specific T cells. **(A)** 5 × 10^6^ naïve OTI cells were adoptively transferred to Tamoxifen-induced SpcCreOVA mice as well as AlbCreOVA, wild-type mice, and non-induced SpcCreOVA mice as controls. Cells were reisolated out of indicated organs 3 days after transfer, stained for the congenic marker Thy 1.1, and analyzed by flow cytometry. To assess proliferation, OTI cells were stained with CFSE before transfer. *n* = 6 out of two independent experiments. Representative histograms of cells are shown for proliferation. **(B)** Experimental scheme of ovalbumin application for *in vivo* activation of adoptively transferred OTI cells. **(C)** OVA expression related to Spc expression was determined by qRT-PCR on day 14 after transfer of indicated numbers of naïve OTI T cells with (gray) or without (white) ovalbumin application; *n* = 3.

We asked if the lack of endogenous T cell response is a consequence of the tolerogenic environment in lung rather than a consequence of central tolerance mechanisms. Thus, we evaluated if adoptively transferred antigen-specific T cells could recognize and eliminate antigen-expressing cells in the lung. To this end, we transferred high numbers (5 × 10^6^) of CFSE-labeled naïve OVA antigen-specific OT-I cells (26) to OVA-induced and non-induced SpcCreOVA mice. As a positive control, we used hepatocyte-specific OVA-expressing AlbCreOVA mice, which activate T cells in liver (24). Three days after transfer, OT-I T cells were reisolated from the lung, liver, peripheral lymph nodes, and spleen. A lack of proliferation and accumulation of transferred T cells were observed in the lung of SpcCreOVA mice, irrespective of antigen expression. In contrast, high proliferation was seen in T cells from AlbCreOVA control mice (Figure 2A). This shows that sterile neoantigen expression in the lung is not recognized by adoptively transferred naïve antigen-specific T cells, despite their expression of a high-affinity T cell receptor.

Next, we investigated if OVA presentation by professional antigen-presenting cells improves the capacity of transferred OT-I cells to detect and clear neoantigen-expressing AECII in the lung. We transferred naïve OT-I T cells to Tamoxifen-induced SpcCreOVA mice and subsequently administered OVA protein either in the lung (intratracheally, i.t.) or peripherally (intraperitoneally, i.p.) (Figure 2B). To assess the cytotoxic capacity of transferred OT-I, OVA expression levels in lungs 2 weeks after transfer were analyzed. We observed that transfer of 5 × 10^6^ OT-I T cells allowed complete clearance of antigen-expressing epithelial cells in the lung irrespective of the route of antigen administration (Figure 2C). Interestingly, when the number of OT-I cells was reduced to 1 × 10^6^, no significant reduction of OVA expression was achieved. This suggests that delivery of neoantigen either into the lung or to the periphery is sufficient to activate T cells. However, to achieve neoantigen reduction, high numbers of antigen-specific T cells are required.

We investigated if vaccination with OVA protein in the periphery can induce endogenous neoantigen-specific T cells in quantities and with qualities sufficient to eliminate antigen-expressing cells in the lungs of SpcCreOVA mice. We applied OVA protein intraperitoneally together with an oil based emulsion adjuvant (Titermax^®^Gold, Sigma). Upon this treatment, 10% of CD8 T cells in lung were found to be specific for OVA. However, no significant reduction of antigen-expressing cells in the lung was achieved by day 14 (Figure S3 in Supplementary Material) suggesting a lack of establishment of cytotoxic T cell response. Similar results were obtained when employing various other peripheral vaccination regimes including DNA immunization (Figures S4 and S5 in Supplementary Material). Together, these results indicate that standard vaccination conditions are not sufficient to raise a functional T cell response targeting the lung tissue.

### TLR Agonists CpG-ODN and LPS Induce Accumulation of Antigen-Specific CD8 T Cells in the Lung

**Figure 3:**
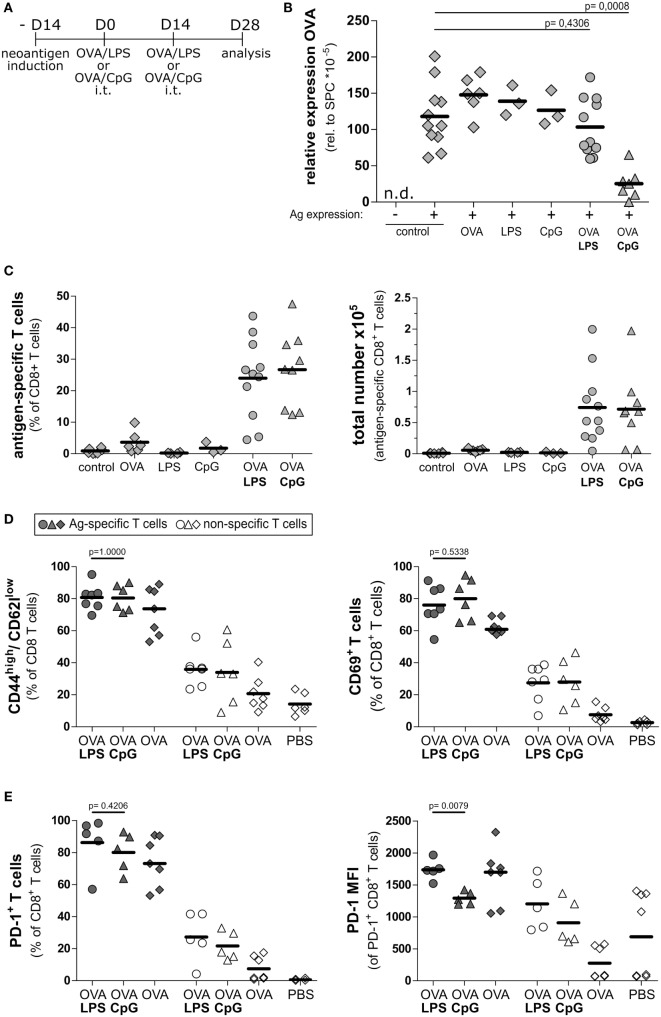
Intratracheal vaccination with CpG/OVA leads to reduction of neoantigen load. **(A)** Experimental scheme for intratracheal vaccinations and analysis of cytotoxic potential on day 28 after vaccination. SpcCreOva mice were induced with Tamoxifen on the day −14 and received two doses of OVA/LPS, OVA/CpG, or single compounds by the intratracheal application at indicated time points. Animals were sacrificed on day 28 after first vaccination. **(B)** OVA expression in the lung of SpcCreOVA mice on day 28 was determined by qRT-PCR and was related to Surfactant Protein C (Spc) expression. n.d., not detected. **(C)** Frequency and a total number of neoantigen-specific CD8^+^ T cells of the indicated groups of mice (all pre-treated with Tamoxifen) were determined by pentamer staining and subsequent flow cytometry analysis. The total number of antigen-specific T cells was calculated based on the total number of isolated cells. Induced but mock-vaccinated mice were used as control. **(D)** CD8 T cells were stained for CD69 and CD44/CD62l and analyzed by flow cytometry. The frequencies of activated T cells (CD69^+^ or CD44^high^/CD62l^low^) was determined in the antigen-specific and the non-specific population of T cells of the same group of mice. **(E)** CD8 T cells were stained for expression of programmed cell death protein 1 (PD-1) and analyzed by flow cytometry. Frequency and median fluorescence intensity (MFI) of PD-1 in antigen-specific and non-specific T cells of the same group of mice are indicated. Circles and triangles indicate OVA/LPS- and OVA/CpG-treated animals, respectively. Diamonds indicate controls as specified. Gray symbols, neoantigen-specific T cells; white symbols, unspecific T cells; *n* = 3–11 out of three independent experiments; *p* values were determined based on Mann–Whitney test.

Recently, it was shown that the specific tolerogenic environment within the lung contributes to the dysfunction of cytotoxic T cells (2, 27). We hypothesized that modulation of the tolerogenic environment by stimulating the pathogen pattern receptors would support T cell functionality in the alveolar space. Recent reports highlighted the relevance of TLR9 agonists in overcoming the tolerogenic environment in liver (28, 29). We asked if stimulation of this pathway would allow modulating the environment in lung. As control, we employed LPS as a well-known adjuvant able to boost the T cell response (30). To induce inflammatory conditions in lung, we intratracheally administered the TLR9 agonist CpG-ODN and the TLR4 agonist LPS, respectively, using concentrations that induced comparable levels of overall inflammation (Figure S6 in Supplementary Material). Both treatments resulted in accumulation of CD11b and MHCII positive cells in lung (Figure S7 in Supplementary Material), which is in line with the recently reported accumulation of these cells in liver (28). We evaluated if these inflammatory conditions would improve the activation and function of endogenous CD8 cells. To this end, we vaccinated Tamoxifen-induced SpcCreOVA mice by intratracheal administration of OVA protein together with either CpG-ODN or LPS. To boost the response, the treatment was repeated after 2 weeks (Figure 3A). Four weeks after first immunization, we assessed the intrapulmonary neoantigen levels as an ultimate result of cytotoxic T cell activity. Strikingly, Tamoxifen-induced SpcCreOVA mice that were treated with the combination of OVA protein and CpG-ODN (OVA/CpG) showed a significant reduction of neoantigen expression in the lung (Figure 3B), indicating ongoing cytotoxic activity in the lung. In contrast, OVA/LPS-treated animals, as well as SpcCreOVA mice treated with single compounds, maintained high levels of antigen expression. To show that the differences in cytotoxicity in OVA/LPS and OVA/CpG treated animals were a consequence of the difference in T cell numbers or activation, we isolated the T cells 4 weeks after first immunization. Interestingly, the frequency of antigen-specific T cells in both OVA/LPS- and OVA/CpG-treated animals was increased to similar levels if compared to single component treated- or mock-treated control groups (Figure 3C). Also, the population of activated neoantigen-specific T cells was elevated to similar levels if compared to the non-specific cells as indicated by the frequency of CD69 and CD44^high^/CD62^low^-expressing cells (Figure 3D). This shows that treatment with both, OVA/CpG and OVA/LPS, leads to a similar increase in activated T cell numbers at this late time point after vaccination. Also, high frequencies of antigen-specific CD8 T cells expressing the exhaustion markers PD-1 (16) (Figure 3E) and Lymphocyte Activation Gen 3 Protein (Lag-3) (31) were found in both OVA/CpG and OVA/LPS mice (Figure S8 in Supplementary Material). Interestingly, in T cells from CpG-ODN/OVA-treated mice, the cellular levels of the exhaustion marker PD-1 was significantly lower than in T cells from mice that received OVA/LPS (Figure 3E).

### OVA/CpG, but Not OVA/LPS Treatment Induces Functional Neoantigen-Specific T Cells in the Lung

**Figure 4:**
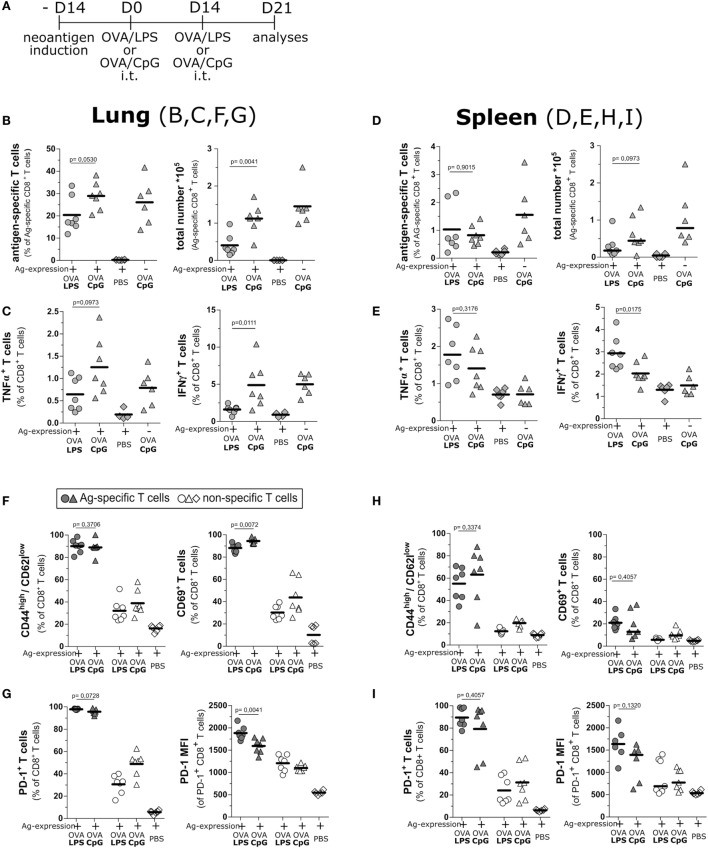
Intratracheal vaccination induces a strong T cell response and maintains production of effector cytokines. **(A)** Experimental scheme for intratracheal vaccinations and evaluation of the activation status of T cells day 21 after vaccination. SpcCreOva mice were induced with Tamoxifen on the day −14 and received two doses of OVA/LPS, OVA/CpG by the intratracheal application at indicated time points. Animals were sacrificed on day 21 after first vaccination. T cells from lung **(B,C,F,G)** and spleen **(D,E,H,I)** were characterized. **(B,D)** Frequency and a total number of neoantigen-specific T cells of the indicated groups of mice were determined by pentamer staining and subsequent flow cytometry analysis. The number of antigen-specific T cells was calculated based on the total number of isolated cells. **(C,E)** TNFα and IFNγ positive CD8 T cells were determined by flow cytometry upon restimulation of isolated T cells with the cognate antigen. **(F,H)** Activation subsets (CD69^+^ or CD44^high^/CD62l^low^) of CD8 T cells from indicated groups was determined by staining for the respective markers and analysis by flow cytometry. **(G,I)** Frequency and median fluorescence intensity (MFI) of programmed cell death protein 1 (PD-1)-positive CD8 T cells were determined by staining and flow cytometry analysis; *n* = 6–7, out of two independent experiment; the Mann–Whitney test was used for statistical evaluation.

To investigate if the notable difference in cytotoxic activity of T cells from OVA/LPS- and OVA/CpG-treated animals was a consequence of differential activation, we analyzed the functionality of T cell response at the peak of induced inflammation, i.e., 1 week after boost vaccination (Figure 4A). Of note, mice treated with OVA/CpG gave rise to a significantly higher number of antigen-specific CD8 T cells in comparison to OVA/LPS-treated mice (Figure 4B). To estimate the cytotoxic potential of these T cells, we assessed cytokine production by intracellular staining after *ex vivo* restimulation with the cognate antigen. In OVA/CpG-treated mice, significantly more antigen-specific T cells accumulated in the lung. These cells expressed higher levels of IFNγ and tended to produce more TNFα in comparison to OVA/LPS-treated mice (Figure 4C). Interestingly, while both vaccination regimes resulted in comparable levels of antigen-specific T cells in the spleen (Figure 4D), CD8 T cells from OVA/LPS-treated mice showed higher levels of effector cytokines if compared to cells from OVA/CpG-treated mice (Figure 4E). This suggests that the CpG-ODN effect is restricted to the lung without manifesting in the periphery.

We further assessed the activation status of the T cells from OVA/CpG and OVA/LPS-treated mice at day 7 post boost vaccination. Higher frequencies of CD44^high^/CD62^low^ neoantigen-specific T cells, in contrast to non-specific cells of both OVA/CpG and OVA/LPS, treated mice were detected (Figure 4F). The same could be observed for CD69 expression on neoantigen-specific and non-specific T cells. At the same time, also the number of cells expressing the exhaustion marker PD-1 was similarly elevated on neoantigen-specific cells from both treatments (Figure 4G). Of note, neoantigen-specific CD8 T cells showed less PD-1 expression per cell after vaccination with OVA/CpG, as indicated by the reduced median fluorescence intensity if compared to OVA/LPS treatment (Figure 4G). In contrast, both treatments gave rise to similar T cell phenotypes in the spleen (Figures 4H,I). Together, this shows that both inflammatory conditions led to the accumulation of a significant number of neoantigen-specific T cells in the lung upon intratracheal vaccination. However, the T cells were better activated upon stimulation of TLR9 as demonstrated by the higher T cell numbers as well as better effector phenotypes of T cells in the early phase of the T cell response.

### Impaired Memory Response to Reinduced Neoantigen Can Be Rescued by Coapplication of CpG-ODN

The previous experiments showed that the impaired T cell response against induced neoantigen expression in AECII could be overcome by vaccination with OVA protein together with triggering the TLR9 signaling pathway.

**Figure 5:**
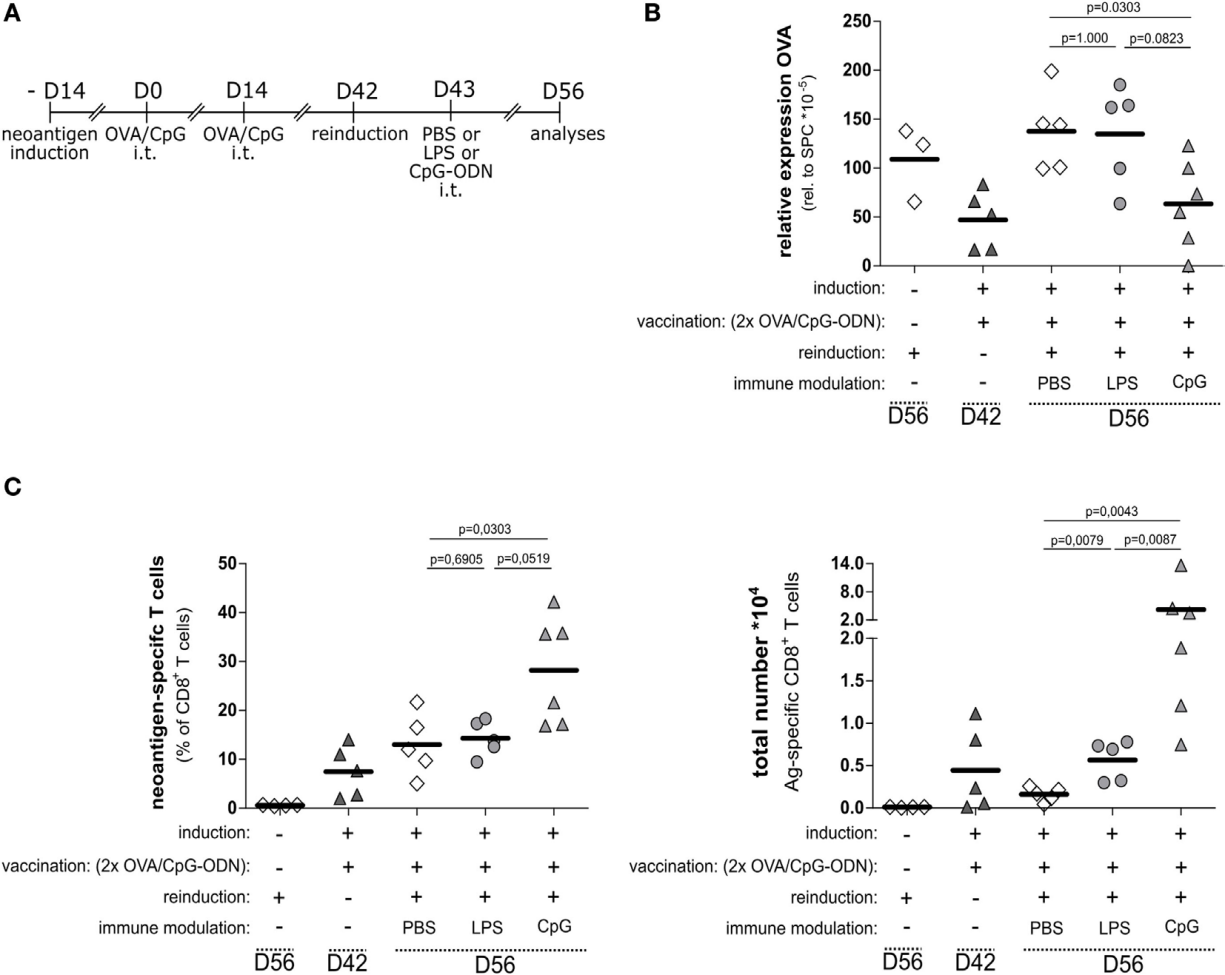
Functional memory T cell response requires immune modulation by the TLR9 pathway. **(A)** Experimental scheme for neoantigen reinduction after prime and boost vaccination with OVA/CpG-ODN. The vaccination scheme as specified in this figure and Figure 4 was employed. On day 42, antigen expression was re-induced by application of 1 mg Tamoxifen; on day 43, LPS, CpG-ODN, or PBS was applied. Analysis of T cells and antigen load was performed on day 56. **(B)** Relative OVA expression in the lung of SpcCreOVA mice with indicated treatments was determined by qRT-PCR **(C)** Frequency and a total number of neoantigen-specific T cells in treated SpcCreOVA mice are presented. The dashed lines indicate time point of sample extraction; *n* = 5–6 out of two independent experiments; statistical evaluation was performed based on the Mann–Whitney test.

We asked if the OVA/CpG-induced T cells would establish memory and develop effector function upon re-induction of neoantigen expression. We investigated this by using the option to re-induce antigen expression by Tamoxifen application. We vaccinated OVA-expressing SpcCreOVA mice with OVA/CpG in the prime/boost protocol as outlined in Figures 3 and 4 and allowed animals to clear the OVA-expressing cells. Four weeks after vaccination, we challenged the mice by applying Tamoxifen, thereby inducing OVA expression in AECII cells (Figure 5A). Antigen re-induction was investigated at different conditions, i.e., in the presence of CpG-ODN, LPS, or without any additional stimulus (PBS control). Two weeks after Tamoxifen application, the animals were sacrificed and the neoantigen load and the number of neoantigen-specific T cells were determined. We observed that the neoantigen load was high and comparable to non-vaccinated SpcCreOVA mice when antigen re-induction occurred upon concomitant intratracheal administration of LPS as well as in the absence of any stimulation (Figure 5B). Also, the number of antigen-specific T cells was comparable in these mice (Figure 5C). However, when we intratracheally applied CpG-ODN during antigen re-induction, we observed a significant reduction of the neoantigen load (Figure 5B), which was accompanied by a high number of neoantigen-specific T cells (Figure 5C).

In summary, while the expansion of endogenous CD8 T cells upon neoantigen expression in the lung is impaired, a strong cytotoxic T cell response can be induced by protein vaccination in the presence of TLR9 stimulation. This immune modulation is also crucial for an efficient memory response toward re-occurring antigen expression.

## Discussion

Immune responses in the lung are considered to be modulated by a specific local environment that balances the high immunogenic challenge in this organ. To investigate the interaction of T cells with antigens in this organ, transgenic mouse models were developed with constitutive antigen expression in a particular cell type of this tissue. By investigating T cells upon interaction with antigen-expressing alveolar cells, an impaired status of T cells could be shown by characterizing the properties of these cells (32, 33). However, these models are inadequate to investigate the cytotoxic functionality of T cells with respect to clearance of antigen load and subsequent tissue regeneration since tissue cells that are eliminated by T cells cannot be replaced by neoantigen-free cells of the same cell type. Thus, these models do not reflect the disease conditions where only part of a cell type will present the antigen and can be substituted by neoantigen-free cells.

With this study, we introduce a new mouse model, SpcCreOVA, which provides a conditional and inducible presentation of the well-described antigen OVA by alveolar epithelial cells type II. As a consequence of the cassette design, feeding of these mice with Tamoxifen induces a genetic switch and expression of OVA in a fraction of AECII cells while a certain number of AECII cells remain uninduced, resulting in a mosaic expression of the antigen. In this regard, this model represents neoantigen expression in a sterile environment of the lung. Similar scenarios are present during the early phase of primary tumor development or in chronic infections of the lower respiratory tract. A hallmark of sterile neoantigen expression is the absence of any antigen-related PAMPs.

By induction of the lung-specific neoantigen expression of SpcCreOVA mice, we observed that endogenous, neoantigen-specific CD8 effector T cells did not develop effector function in the lung and could not reduce the neoantigen load within 15 weeks. Thus, the immune system tolerates or even ignores the expression of a neoantigen in the context of the lung. Even when we transferred neoantigen-specific T cells to these animals, no reduction of neoantigen on AECII was detected, which is in line with the previous reports (34). Remarkably, providing the cognate antigen to the site of antigen expression or the periphery, led to *in vivo* activation of transferred antigen-specific T cells and was sufficient to induce effector functions. This indicates on one hand that neoantigen presentation by APCs is not taking place upon activation of neoantigen expression by AECII. On the other hand, clearance of neoantigen-expressing cells by *in vivo* activated, transferred OT-I T cells confirms sufficient neoantigen presentation by AECII. Still, a high number of neoantigen-specific T cells had to be transferred to achieve clearance of neoantigen-expressing AECII cells. This indicates that the regulatory mechanism in the lung can be overcome by a high amount of antigen-specific T cells.

We thus hypothesize that the tolerogenic environment in the lung as such blocks CD8 T cell responses and thereby impairs clearance of antigen-expressing alveolar epithelial cells type II. Such conditions could contribute to the establishment of a niche for primary tumor development [e.g., secondary lung cancer (2)] or chronic viral infections.

We modulated the immune suppression in the lower respiratory tract by mimicking infections using TLR agonists. Interestingly, we find that stimulation of TLR4 or TLR9 helped to improve the T cell phenotype. Concordantly, by using the TLR agonists LPS or CpG-ODN in intratracheal therapeutic OVA antigen vaccination, we observed an expansion of endogenous, neoantigen-specific T cells in the lung. Moreover, the neoantigen-specific CD8 T cells but not the unspecific T cells on the site of neoantigen expression were highly activated, indicating a specific response in the lung, rather than an unspecific inflammation. At the same time, also the expression of exhaustion marker PD-1 and Lag3 was increased on neoantigen-specific T cells. Increased expression of PD-1 correlates with the lack of cytotoxic function of CD8 T cells (35). Strikingly, T cells induced by CpG/OVA showed less PD-1 expression, in comparison to T cells expanded by OVA/LPS treatment. Moreover, they developed a potent cytotoxic T cell activity, indicated by the reduction of neoantigen expression in the lung after therapeutic vaccination and the expression of effector cytokines after *ex vivo* restimulation. Together, this shows that the TLR9 agonist CpG-ODN and the TLR4 agonist LPS both promote the proliferation and recruitment of T cells to the lung after intratracheal vaccination. However, only triggering the TLR9 can promote the development of functional T cell responses that is capable of reducing the neoantigen load. In this regard, the SpcCreOVA model lends itself as a tool to determine the immunogenic potential of other mucosal adjuvants like c-di-AMP od c-di-GMP (36, 37) allowing the evaluation of the cytotoxic potential of the induced immune response to sterile neoantigen expression in the lower respiratory tract.

The observation that stimulation of TLR9, but not stimulation of TLR4, can promote the cytotoxic activity of T cells was not expected given that stimulation of TLR4 and TLR9 receptors initiate a similar downstream cascade and converges in NFκB-mediated activation of cytokine expression. However, these pathways are not redundant. While TLR9 signaling is mediated exclusively *via* MyD88, activation of the TLR4 pathway signals *via* both MyD88 and TRIF. This dual signaling results in the induction of a particular set of genes that is specific for the TLR4 pathway, but that is not induced by individual triggering of MyD88 or TRIF. In addition, TLR4 signaling has been shown to have an inhibitory effect on the expression of certain genes that are induced when by MyD88 or TRIF are activated individually (38). Therefore, certain crucial components induced by TLR9 might be downregulated upon TLR4 stimulation, which might explain the different outcome using these two immune modulators.

Although we used conditions in which the two TLR agonists induced comparable overall inflammatory burden treatment with CpG treatment supports both, more efficient expansion of neoantigen-specific T cells and an increase in the functionality. This indicates that immunomodulation based on TLR9 stimulation is quantitatively and qualitatively influencing CD8 T cell responses in the lung.

While there are differences in the TLR9 expression pattern in between mouse and man, both species express TLR9 in the lung tissue (39). In humans, TLR9-expressing pDCs have been implicated to serve a protective role (40). Moreover, recent studies indicate that under certain conditions also non-immune cells such as Type II AECs, airway smooth muscle cells, airway epithelial cells, and fibroblasts induce TLR9 expression (41). Increased TLR expression could be as well identified in histological analysis of diseased human lungs (39). To estimate the translational value of our findings, further analysis of the consequences of TLR9 stimulation on the cellular and humoral responses is required.

Strikingly, the successful reduction of neoantigen-expressing AECII by T cells induced with OVA/CpG vaccination does not lead to a long-term protection against re-occuring neoantigen expression. Mice that successfully cleared the antigen-expressing cells upon therapeutic vaccination with OVA/CpG still depended on repeated administration of CpG-ODN during antigen relapse. By reinduction of neoantigen expression, we could show that the low number of remaining neoantigen-specific CD8 T cells, 4 weeks after sufficient therapeutic vaccination, could not develop effector function. This indicates a transient CpG-ODN effect and confirms the need of immune modulation to induce potent T cell responses against neoantigens presented in the tolerogenic environment of the lung. Thus, immune modulation by intratracheal CpG-ODN, but not by LPS, after relapse of neoantigen expression can rescue impaired memory T cells in the tolerogenic environment of the lung.

Previously, it was shown that intravenous application of TLR9 agonist induces clusters of professional antigen-presenting cells in the liver, which support CD8 T cell expansion in viral infection (28). In line with this, we recently showed that CpG-ODN rescues impaired CD8 T cell effector function in the liver (29). In this study, we show that intratracheal application of CpG and LPS induces clusters of CD11b/MHCII cells also in lung; however, only CpG supports a potent T cell response. While the molecular mechanisms underlying the positive effect of TLR9 stimulation in lung remains to be elucidated, our results indicate that CpG-ODN TLR ligands are suitable candidates for the immunomodulatory treatment against neoantigen-expressing cells. In this regard, the transgenic mouse model developed for this study might be of interest for the rational development of therapeutic vaccines or adjuvants. Importantly, such immune modulation on the site of neoantigen expression should be considered for cancer treatment and therapeutic vaccinations.

## Ethics Statement

This study was carried out in accordance to the national and local guidelines. The protocol has been approved by the local authorities (Niedersächsisches Landesamt für Verbraucherschutz und Lebensmittelsicherheit’, Dezernat 33). An ethics committee is not required according to the national and local guidelines.

## Author Contributions

Concept and design: MR and DW. Experiments and procedures: MR and MC. Critical discussion of data and writing the manuscript: MR, MC, HH, and DW. All authors read and approved the manuscript.

## Conflict of Interest Statement

The authors declare that the research was conducted in the absence of any commercial or financial relationships that could be construed as a potential conflict of interest.
