# Supplementary material for: CpG-ODN Facilitates Effective Intratracheal Immunization and Recall of Memory against Neoantigen-Expressing Alveolar Cells
Source: Front Immunol. 2017 Sep 29;8:1201. doi: 10.3389/fimmu.2017.01201 (PMC5630691; doi:10.3389/fimmu.2017.01201)
Supplement: Supplementary file 1 [file Presentation_1.pdf]

Supplementary Figures

Figure S1

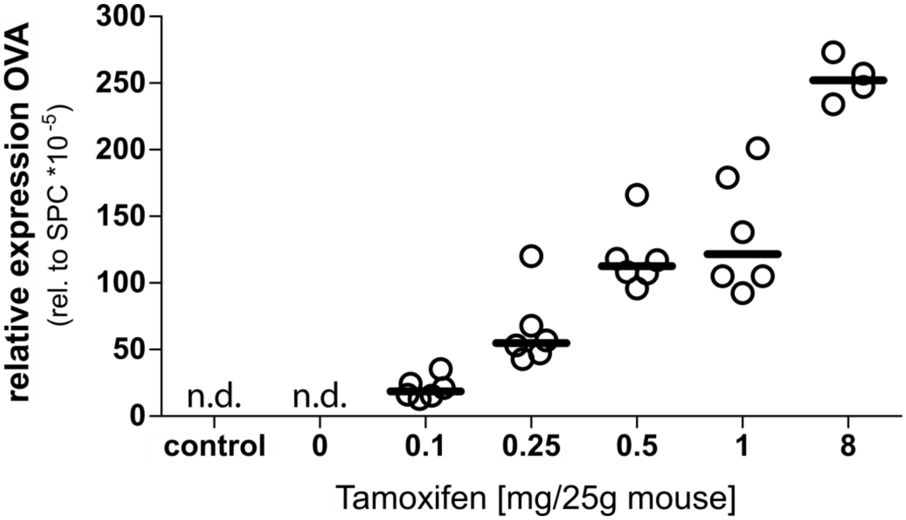

Figure S1: Neoantigen load in the lung correlates with the amount of Tamoxifen

The expression levels of OVA neoantigen in lungs of SpcCreOVA mice were determined by qRT-PCR two weeks after application of indicated amounts of Tamoxifen. The expression level of OVA was related to the expression of Surfactant Protein C (Spc). Results of individual mice as well as the median expression are shown from two independent experiments. n.d.: not detected. Wild type mice were used as control.

Figure S2

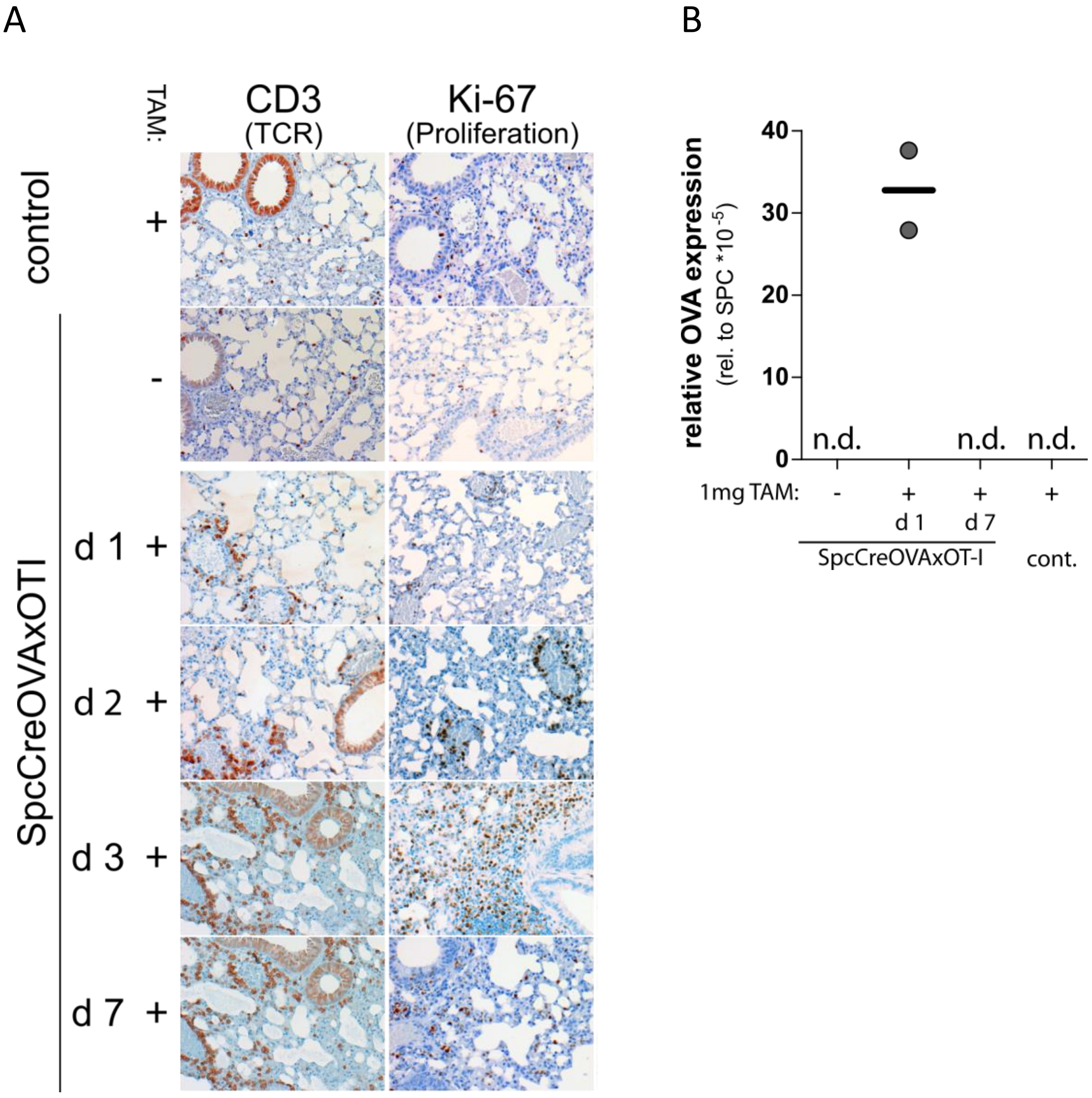
A
B
TAM:
CD3
(TCR)
Ki-67
(Proliferation)
control
+
-
SpcCreOVAxOTI
d 1 +
d 2 +
d 3 +
d 7 +
relative OVA expression
(rel. to SPC *10-5)
40
30
20
10
0
n.d.
n.d.
n.d.
1mg TAM:
-
+
+
+
d 1
d 7
SpcCreOVAxOT-I
cont.

Figure S2: T cell activation and cytotoxic activity after induction of neoantigen expression in SpcCreOVAxOT-I

(A) SpcCreOVAxOT-I mice were fed with 1mg Tamoxifen. On indicated time points, lung slices were fixated, embedded in paraffin and stained with αCD3 and αKi-67 and visualized by 3,3'-Diaminobenzidine staining (brownish spots). Shown are representative slices from total 3 mice per group. Controls are RosaOVA animals without Cre. **(B)** SpcCreOVAxOT-I mice were fed with 1mg Tamoxifen. At indicated time points, lung sections were collected and mRNA was isolated. Results of qRT-PCR for OVA expression were related to Spc. n.d.: not detectable, control= littermates without Cre; n=2 per group

Figure S3

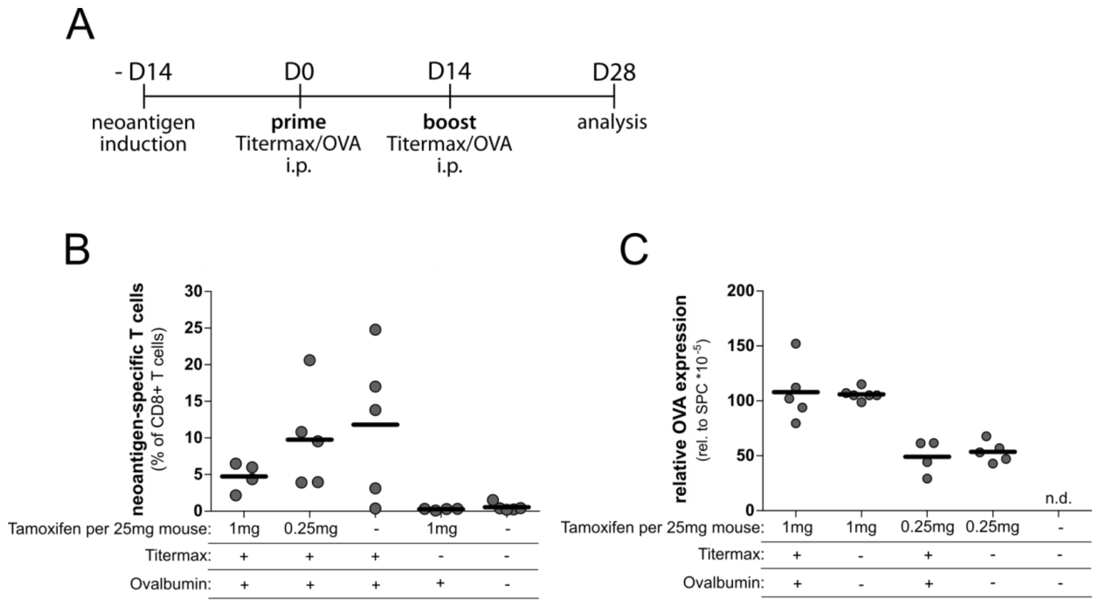

Figure S3: Intraperitoneal vaccination in induced SpcCreOVA mice indeced neoantigen-specific CD8 T cell but could not deliver effector function.

(A) Experimental scheme for the therapeutic vaccination of induced SpcCreOVA with Titermax gold (Sigma) and 25-50µg Ovalbumin intraperitoneal **(B)** Frequency of neoantigen-specific CD8 T cells in the lung two weeks after treatment **(C)** Neoantigen load by qRT PCR in the lung after treatment. n= 4-6 out of two independend experiments

Figure S4

Prophylactic DNA vaccination

A

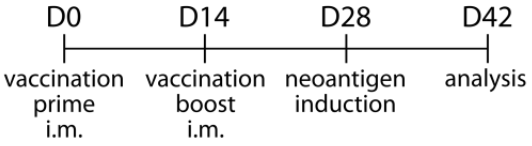

B

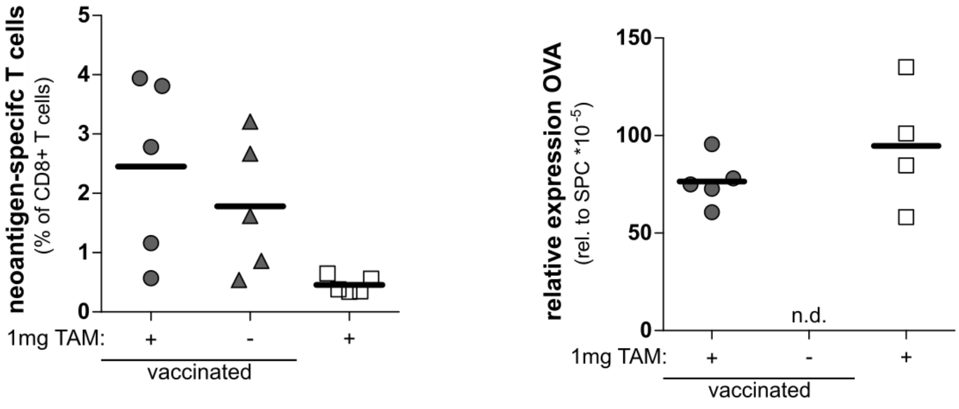

Figure S4: Preventive plasmid immunization improved expansion of endogenous, neoantigen-specific T cells

(A) Scheme for the preventive vaccination and subsequent neoantigen induction in the lung of SpcCreOVA mice. **(B)** Endogenous neoantigen-specific T cells were isolated two weeks after feeding of Tamoxifen and stained with pentamer. The relative OVA expression in the lung was investigated by qRT-PCR two weeks after neoantigen induction. n=4-5 obtained from one experiment

Figure S5

Therapeutic DNA vaccination

A

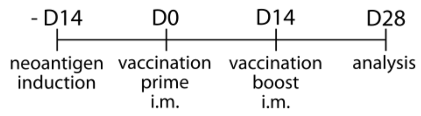

B

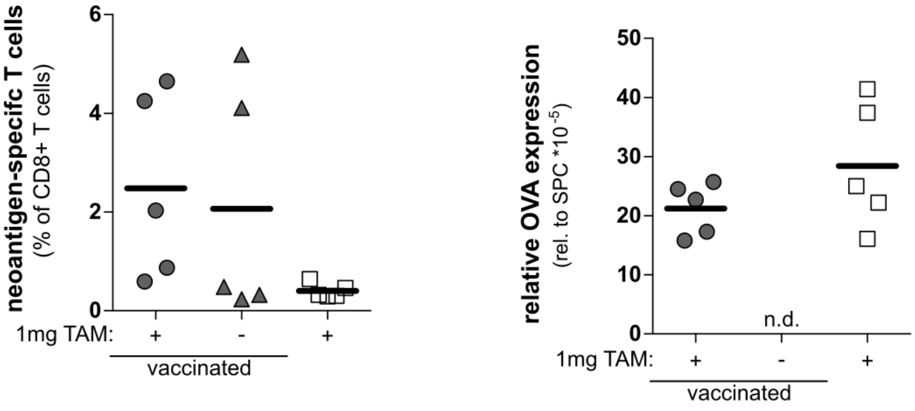

Figure S5: Therapeutic pCI-OVA vaccination enhanced the number of neoantigen-specific T cells

(A) Scheme for therapeutic vaccination regime against OVA neoantigen in the lung of SpcCreOVA mice. **(B)** Endogenous neoantigen-specific T cells were stained with pentamer specific for OVA two weeks after vaccination. The relative OVA expression in the lung was investigated by qRT-PCR two weeks after neoantigen induction. n.d. = not detectable; n=5, lines indicate the mean of expression.

Figure S6

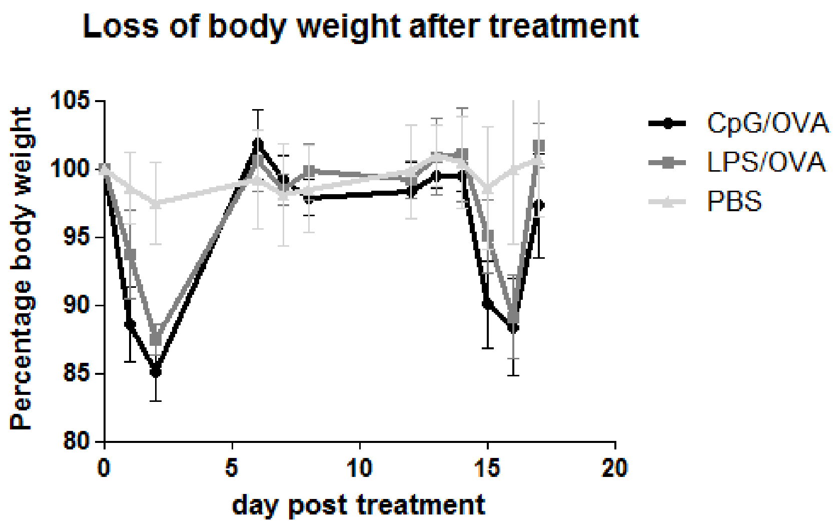

Figure S6: Loss of body weight upon intratracheal vaccination

Tamoxifen induced SpcCreOVA were vaccinated intratracheally on day 0 and 14 with CpG/OVA, LPS/OVA or PBS as indicated. Body weight on day 0 before treatment was set to 100%. N=3. One representive experiment out of 6 independent experiments is depicted

Figure S7

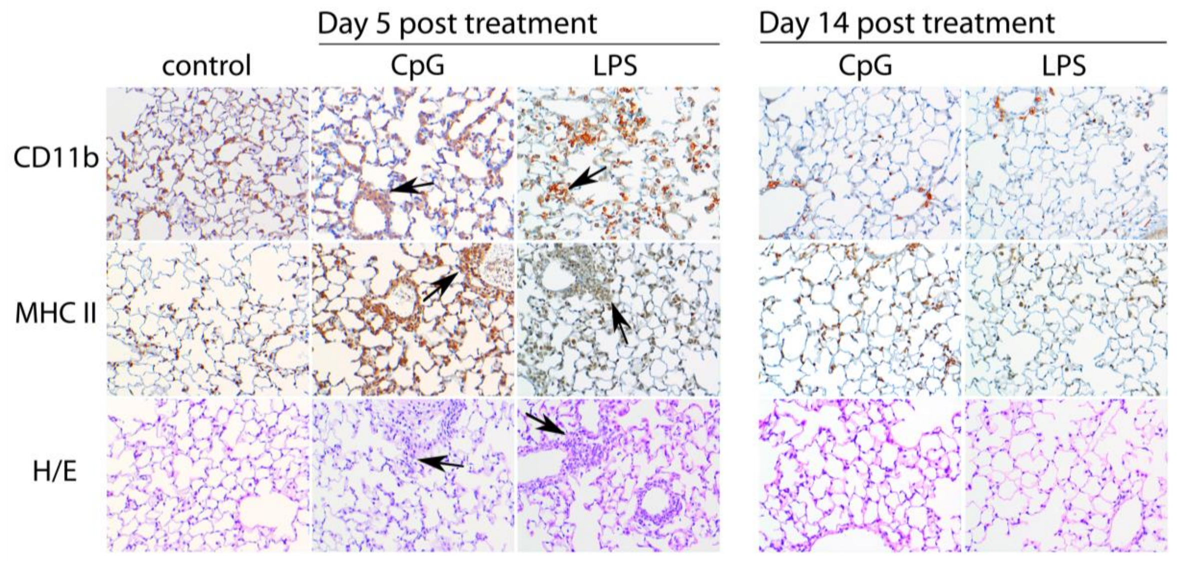

Figure S7: Histology of lung after application of TLR-agonists

Lung slices of Tamoxifen induced SpcCreOVA mice at indicated time points upon treatment with indicated immune modulators (CpG-ODN, LPS or PBS-treated animals for control) were immunohistochemically staining for CD11b and MHC II expression. In addition, hematoxylin and eosin staining (H/E) was performed. Representative pictures are depicted from two independent experiments with 2-4 mice per group.

Figure S8

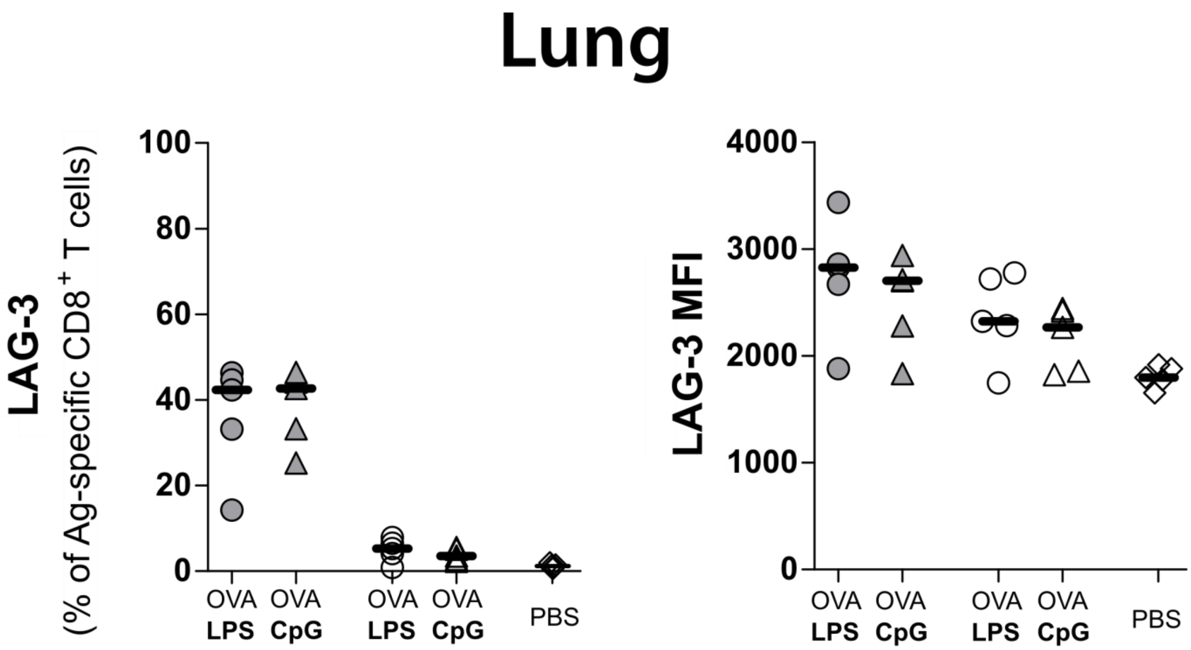

Figure S8: Elevated Lag-3 expression on neoantigen-specific CD8 T cells in the lung

SpcCreOva mice were induced with Tamoxifen on day -14 and received two doses of OVA/LPS or OVA/CpG. Animals were sacrificed on day 14 after treatment. CD8 T cells were stained for expression of Lag-3 and analyzed by flow cytometry. Frequency and median fluorescence intensity (MFI) of Lag-3 in neoantigen specific and non-specific T cells of the same group of mice are depicted. Circles and triangles indicate OVA/LPS and OVA/CpG treated animals, respectively. Diamonds indicate controls as specified; Grey symbols= neoantigen-specific T cells; white symbols = unspecific T cells; n= 4-5 out of 2 independent Experiments
